# MiR-107 confers chemoresistance to colorectal cancer by targeting calcium-binding protein 39

**DOI:** 10.1038/s41416-019-0703-3

**Published:** 2020-01-10

**Authors:** Yu Liang, Danxi Zhu, Lidan Hou, Yu Wang, Xin Huang, Cui Zhou, Liming Zhu, Yingying Wang, Lei Li, Yan Gu, Meng Luo, Jianhua Wang, Xiangjun Meng

**Affiliations:** 1 Department of Gastroenterology, Shanghai Ninth People’s Hospital, Shanghai Jiao Tong University School of Medicine, Shanghai, 200011 China; 2 Department of Biochemistry and Molecular & Cell Biology, Shanghai Jiao Tong University School of Medicine, Shanghai, 200025 China; 3 Department of General Surgery, Shanghai Ninth People’s Hospital, Shanghai Jiao Tong University School of Medicine, Shanghai, 200011 China; 4 Cancer Institute, Fudan University Shanghai Cancer Center, Shanghai, 200032 China

**Keywords:** Molecular medicine, miRNAs

## Abstract

**Background:**

Chemoresistance remains a critical event that accounts for colorectal cancer (CRC) lethality. The aim of this study is to explore the ability of dichloroacetate (DCA) to increase chemosensitivity in CRC and the molecular mechanisms involved.

**Methods:**

The effects of combination treatment of DCA and oxaliplatin (L-OHP) were analysed both in vitro and in vivo. The DCA-responsive proteins in AMPK pathway were enriched using proteomic profiling technology. The effect of DCA on CAB39–AMPK signal pathway was analysed. In addition, miRNA expression profiles after DCA treatment were determined. The DCA-responsive miRNAs that target CAB39 were assayed. Alterations of CAB39 and miR-107 expression were performed both in vitro and on xenograft models to identify miR-107 that targets CAB39–AMPK–mTOR signalling pathway.

**Results:**

DCA increased L-OHP chemosensitivity both in vivo and in vitro. DCA could upregulate CAB39 expression, which activates the AMPK/mTOR signalling pathway. CAB39 was confirmed to be a direct target of miR-107 regulated by DCA. Alterations of miR-107 expression were correlated with chemoresistance development in CRC both in vitro and in vivo.

**Conclusion:**

These findings suggest that the miR-107 induces chemoresistance through CAB39–AMPK–mTOR pathway in CRC cells, thus providing a promising target for overcoming chemoresistance in CRC.

## Introduction

Colorectal cancer (CRC) is the third most commonly diagnosed cancer in males and the fourth most common cancer in females; it is also the second leading cause of cancer death (9.2% of all the cancer deaths) according to the Global Cancer Statistics for 2018.^1^ Although a growing number of targeted drugs have become available to treat metastatic colorectal cancer, the 5-year relative survival rate for patients with CRC is only 65%. Chemotherapy remains the main treatment for stage IV cancers.^2^ Therefore, elucidating the mechanisms of chemoresistance and seeking new optimal clinical strategies are urgently needed.

The metabolic features of malignant tumour cells are distinctly and closely related to tumour development, invasion and metastasis. Therefore, the development of specific small molecules targeting cancer metabolism has become a potential strategy. Dichloroacetate (DCA) is a drug used in treating mitochondrial metabolic disorders, such as lactic acidosis. Recently, DCA has been reported to have anticancer effect because it can serve as an inhibitory molecule of pyruvate dehydrogenase kinase (PDK).^3^ Although DCA has been studied in multiple cancers including prostate and breast cancer,^4,5^ the mechanism of how DCA exerts its anticancer effects remains unclear.

Oxaliplatin (L-OHP), as a platinum chemotherapeutic drug, is widely used for clinical colorectal cancer treatment. However, drug resistance is one of the major challenges for clinical chemotherapy failure. It has been reported that the AMPK and mTOR, master regulators of cellular energy and insulin signalling, highlight the association between the metabolic alterations and chemoresistance, and provide ideal targets for intervention.^6–8^ Calcium-binding protein 39 (CAB39), a scaffold protein of liver kinase B1 (LKB1), is a key regulatory factor upstream of AMPK/mTOR pathway.^9^ Although the mechanism of CAB39 in regulating the conformation of STE20 kinases and activating catalytically competent protein kinases has been identified, the roles of CAB39 in CRC have not been discussed.^10^

MicroRNAs (miRNAs) are a family of small, noncoding RNA molecules that play important roles in multiple biological processes, including cell proliferation, apoptosis and organ development, in different cancers.^11^ A large body of evidence suggests that miRNAs are closely associated with the development of chemoresistance in cancer cells,^12–17^ indicating that miRNAs might be potential targets for cancer treatment. Previous studies showed that miR-107 expression is significantly higher in gastric cancer tissues than in adjacent normal tissues, and downregulation of miR-107 expression suppresses tumour cell proliferation, migration and invasion.^18–20^ Similarly, miR-103/107-mediated inhibition of DAPK and KLF4 was reported to promote colon cancer metastasis.^21^ However, the clinical significance and detailed mechanisms of its involvement in chemoresistance still remain to be fully clarified.

Here, we reported that DCA treatment induced CAB39 expression through its direct target miR-107 and subsequently modulated the activities of AMPK/mTOR signal pathway, which is involved in overcoming CRC chemoresistance to L-OHP.

## Methods

### Cells and reagents

The human colon cancer cell lines HCT-8 and LoVo, and the HEK293T cell line were purchased from the American Type Culture Collection (ATCC, Manassas, VA, USA). The chemoresistant cell line HCT-116/L-OHP and its parental HCT-116 cell lines were kindly provided by Dr. Lin from Cancer Hospital Chinese Academy of Medical Sciences. The HCT-116/L-OHP cell line was insensitive to L-OHP that was derived from its parental cell line by serial passaging in the presence of increasing L-OHP concentrations (continuous exposure) and it was maintained in a medium containing 4 μg/ml L-OHP. Cell lines were cultured in Dulbecco’s modified Eagle’s medium or RPMI-1640 medium containing 10% foetal bovine serum (FBS), 100 U/mL penicillin and 100 μg/mL streptomycin in a 37 °C, 5% CO_2_ humidified incubator. The cell lines were characterised by the Genetic Testing Biotechnology Corporation (Suzhou, China) by using short tandem repeat (STR) markers. L-OHP was purchased from Jiangsu Heng Rui Pharmaceuticals Co. Ltd. (Jiangsu, China). DCA was purchased from Sigma-Aldrich Co. Ltd. (MO, USA) and a 20 mM dosage was applied in vitro.

### Oligonucleotide transfection

Small interfering RNAs targeting CAB39, miRNA mimics, inhibitors and their corresponding controls were synthesised by GenePharma (Shanghai, China). Oligonucleotide transfection was performed with Lipofectamine 3000 (Invitrogen, CA, USA) at a final concentration of 50 (mimics and siRNAs) or 100 nmol/L (inhibitors). Cells were harvested for the assays 24 or 48 h after transfection.

### RNA extraction, reverse transcription, miRNA microarray and quantitative real-time PCR

Total RNA was extracted from tissues or cells with TRIzol Reagent from Life (CA, USA) according to the manufacturer’s protocol. cDNA was synthesised using a PrimeScript RT Reagent Kit from TaKaRa (Tokyo, Japan). The microarray assay was performed by Kangchen Bio-tech (Shanghai, China) with three replicates of HCT-116 cells treated with 5, 10 or 20 mM DCA for 12, 24 or 48 h. Real-time PCR was performed by using TaKaRa premix Ex Taq 420 A (Tokyo, Japan). β-actin and U6 small nuclear RNA were used as loading controls. The original data of miRNA microarray were uploaded to GEO database (GSE125309).

### Vector transfection

The 3′UTR of CAB39, harbouring either the wild or mutant form of the miR-107 binding sequence, was inserted downstream of the stop codon of Renilla luciferase in the pRL-CMV vector (Promega, WI, USA), while the pGL-3 plasmid (Firefly) was co-transfected as an endogenous control for normalisation. The pENTER-CAB39 plasmid and its control were purchased from Vigene (Shandong, China). A miR-107 Sponge and its control plasmid, as well as the packaging and envelope plasmids were purchased from Yile (Shanghai, China). Lentivirus particles were harvested 48 h after lentiviral vector transfection of the packaging and envelope plasmids into human embryonic kidney 293T cells using Lipofectamine 3000 reagent. Cells were infected with recombinant lentivirus plus 6 μg/mL polybrene from Sigma-Aldrich (MO, USA).

### Luciferase assay

Human HCT-116 cells were cultured in 96-well plates and co-transfected with 10 ng of pRL-CMV-CAB39 3′UTR and 5 pmol miR-107 mimics or negative control. After a 48-h incubation, firefly and renilla luciferase activities were measured by using the Dual-Luciferase Reporter Assay System from Promega (WI, USA). The data are shown as the ratio between renilla and firefly fluorescence activities.

### Western blot analysis

For Western blot analysis, 20 μg of total protein lysates (RIPA lysis buffer) were subjected to sodium dodecyl sulfate-polyacrylamide gel electrophoresis. The primary antibodies used in this study were as follows: anti-CAB39 (1:10,000, Abcam, Cambridge, UK), anti-p-AMPK (1:1000, CST, MA, USA), anti-AMPK (1:1000, CST, MA, USA), anti-p-mTOR (1:1000, CST, MA, USA), anti-mTOR (1:1000, CST, MA, USA), anti-β-actin (1:2000, Arigo, Taiwan) and anti-α-tubulin (1:2000, Proteintech, Wuhan, China). Secondary antibodies were purchased from Sungene (Tianjin, China). The proteins were detected with Millipore ECL (MA, USA).

### Cell viability assays

Cell proliferation was determined by using a Cell Counting Kit-8 (CCK-8) assay from Dojindo (Japan). Briefly, the cells were seeded in 96-well plates at 1 × 10^4^ cells/well. After incubation for 24 h, the medium was removed and replaced with fresh medium that contained varying concentrations of L-OHP or/and 20 mM DCA for 24 h. Then, 110 μl of mixture medium containing 10 μl of CCK-8 reagent was placed in each well, and the cells were incubated for an additional further 2 h. Absorbance was read at 450 nm by using an enzyme microplate reader. The inhibition ratio was calculated by using the following formula: (OD^control^– OD^treated^)/ OD^control^.

### Apoptosis assay

Apoptosis was measured 48 h after transfection by using an Annexin V/fluorescein isothiocyanate (FITC) apoptosis detection kit or Annexin V/PE Apoptosis Detection Kit I (BD Biosciences, USA). Briefly, cells were trypsinised, washed, stained with antibody in darkness for 15 min at room temperature and then measured by flow cytometry. Each experiment was performed in triplicate.

### Colony formation assay

Cells were seeded in six-well culture plates at a density of 2500 cells/well in the medium containing different concentrations of drugs for 48 h. After incubation in fresh medium for 10–14 days at 37 °C, the colonies were washed twice with PBS and stained with a haematoxylin solution. The colonies composed of more than 50 cells in a well were counted under a microscope. All the experiments were repeated at least three times.

### Xenografts in nude mice

Eight-week-old male nude mice (BALB/c) were purchased from JRDun Biotechnology (Shanghai, China), and were maintained under specific pathogen-free, 12-h light–dark cycle (room temperature 22.5 ± 0.2 °C, humidity 40–60%) conditions and the animals had free access to feedstuff and water. To ameliorate the suffering of mice observed throughout experimental studies, mice were euthanised by CO_2_ inhalation. Animal care and experiments were approved by the Ethical Committee of the Ninth People's Hospital Affiliated with the Medical College of Shanghai Jiao Tong University (HKDL[2018]379) and were in accordance with the Provision and General Recommendation of Chinese Experimental Animals Administration Legislation.

HCT-116/L-OHP cells were grown in culture and then detached by trypsinisation, washed and resuspended in Hanks balanced salt solution (HBSS). Next, 0.2 mL of HBSS with cells (5 × 10^6^) was injected subcutaneously into the nude mice to initiate tumour growth. When the tumours reached an average size of 50 mm^3^, the mice were randomised into six groups (n = 6 per group): 1) control, 2) DCA alone (DCA^a^), 3) oxaliplatin alone (L-OHP), 4) DCA^a^ + L-OHP, 5) DCA alone (DCA^b^) and 6) DCA^b^ + L-OHP. DCA^a^: DCA (0.075 g/l) was added to the drinking water; DCA^b^: DCA was injected intratumourally at a concentration of 50 mg/kg. L-OHP was injected intraperitoneally every 2 days at a concentration of 5 mg/kg. Tumour volume was calculated by using the following formula: volume (mm^3^) = (width)^2^ × length × 0.5. Tumour volume and body weight were measured every 3 days. Five weeks after treatment, the mice were sacrificed and weighed, and the tumours were excised and weighed.

HCT-116/miR-107-overexpressing cells (5 × 10^6^) or HCT-116/vector cells were injected subcutaneously into the right flank of the nude mice (BALB/c). The tumour size was measured with a calliper every 3 days after tumour formation. When the tumours were approximately 50 mm^3^ in size, both groups began to receive intraperitoneal injections of L-OHP (5 mg/kg) every other day. After 4 weeks of administration, the mice were sacrificed and photographed, and the tumours were removed and weighed.

### Immunohistochemistry

Paraffin sections prepared from in vivo experiments were used for immunohistochemistry assays to detect protein expression levels of CAB39, p-AMPK and p-mTOR expression level. The indirect streptavidin-peroxidase method was used according to the manufacturer’s instructions. The antibodies used were rabbit anti-CAB39 (ab51132, Abcam), anti-p-mTOR (ab109268, Abcam) and anti-p-AMPK (orb5692, Biorbyt).

### Human specimens

Human primary CRC tissues and matched normal tissues (at least 10 cm from the tumour margin, verified to be free of tumour) obtained from surgical resection were snap-frozen immediately in liquid nitrogen. Twenty-six tumour tissues and twenty-four normal tissues were collected from Shanghai Ninth People’s Hospital after obtaining informed consent. All procedures were approved by the Ethical Committee of the Ninth People's Hospital Affiliated to the Medical College of Shanghai Jiao Tong University (SH9H-T169-1) and were in accordance with the Declaration of Helsinki.

### Statistical analysis

The data are expressed as the mean ± standard deviation from one representative experiment of at least three independent experiments. Student’s *t* test was used to compare the differences between two groups unless otherwise noted. A paired *t* test was used to analyse miR-107 and CAB39 mRNA levels in human samples. The Spearman method was performed to analyse correlations. *P* < 0.05 was considered to indicate a statistically significant difference. Statistical analyses were conducted by using GraphPad Prism 5.0 (CA, USA).

## Results

### DCA enhances L-OHP chemosensitivity both in vitro and in vivo

**Fig. 1:**
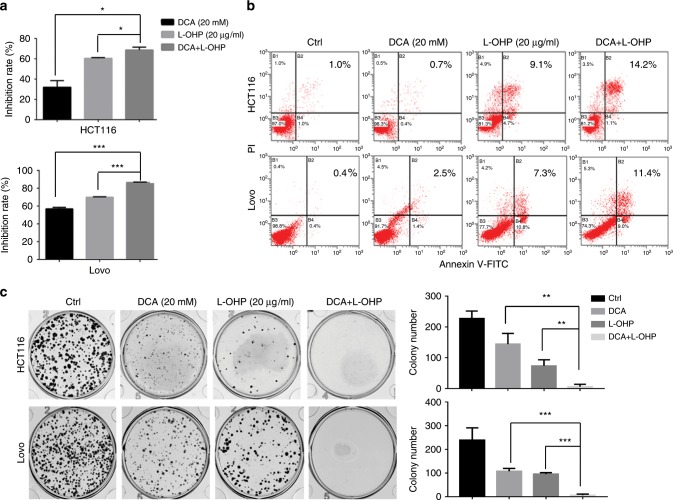
The combination effect of DCA and L-OHP in CRC cells. HCT-116 and LoVo cells were treated with 20 mM DCA or 20 μg/ml L-OHP for 48 h. **a** The inhibition rate was measured by CCK-8 assay. **b** Cell apoptosis was measured by flow cytometry. **c** Colony formation assay was determined by crystal violet staining. Each experiment encompassed three replicates. *P < 0.05, **P < 0.01, ***P < 0.001. A representative experiment of three independent experiments is shown.

DCA, as a known regulator of PDK, is thought to have synergic effects with chemotherapy drugs in suppressing cancer cell growth. Here, we examined the synergistic effects of DCA combined with L-OHP in CRC cell lines (HCT-116 and LoVo). The inhibition rate was higher in the drug combination group (DCA and L-OHP) than in the DCA or L-OHP group alone (Fig. 1a). Apoptosis was higher in those cells treated with the combination of DCA and L-OHP than cells treated with DCA or L-OHP alone (Fig. 1b). Meanwhile, the effects of colony formation capacity of those cells were also similarly observed (Fig. 1c).

**Fig. 2:**
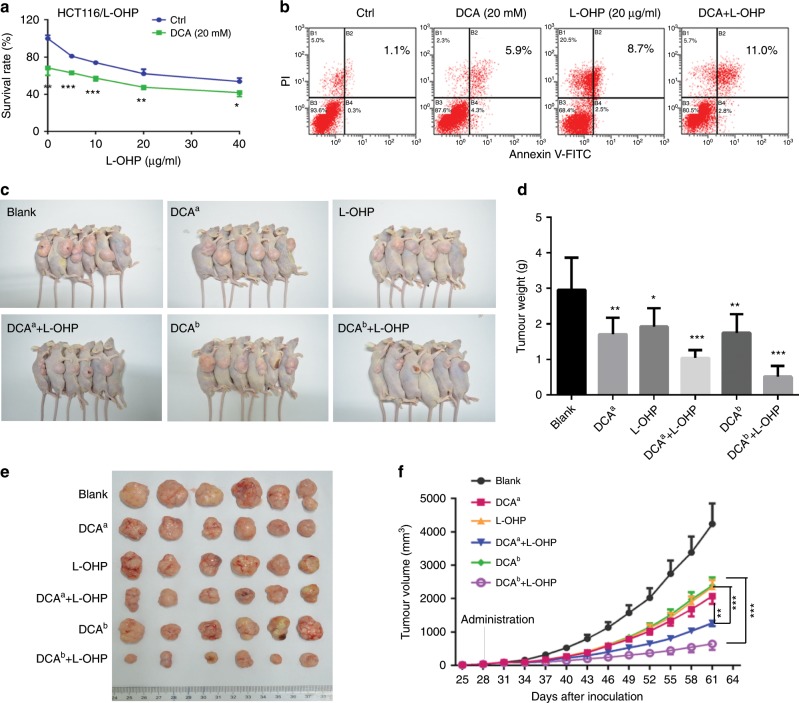
DCA increases L-OHP chemosensitivity in vivo. **a** HCT-116/L-OHP cells were treated with different concentrations of L-OHP with or without 20 mM DCA for 48 h. The survival rate was measured by CCK-8 assay. **b** HCT-116/L-OHP cells were treated with 20 mM DCA or 20 μg/ml L-OHP for 48 h. Cell apoptosis was measured by flow cytometry. Each experiment encompassed three replicates. *P < 0.05, **P < 0.01, ***P < 0.001. **c**–**f** The in vivo effects of DCA alone or in combination with L-OHP in the xenograft model, *n* = 6/group. DCA^a^: DCA (0.075 g/l) was added to the drinking water, DCA^b^: DCA was intratumourally injected at a concentration of 50 mg/kg. **c** The photograph of sacrificed mice. **d** The tumour weights were measured. **e** Representative photograph of tumours. **f** Tumour volume was measured and tumour growth curves were plotted. *n* = 6, *P < 0.05, **P < 0.01, ***P < 0.001.

To further confirm that DCA can regulate the chemoresistance of CRC cells to L-OHP, HCT-116/L-OHP cell line that is insensitive to L-OHP is used. DCA treatment significantly decreased the survival rate of HCT-116/L-OHP cells upon treatment with different concentrations of L-OHP (Fig. 2a). Apoptosis was higher in the combination group than DCA and L-OHP-alone groups of HCT-116/L-OHP cells (Fig. 2b). Based on these findings, we then examined the combination effect of DCA and L-OHP in vivo. The volume and weight of tumours were significantly lower in mice treated with both DCA and L-OHP than the respective controls treated with the single drugs and quantification analyses confirmed the results (Fig. 2c–f). These results suggest that DCA could increase L-OHP chemosensitivity.

### Reduction of CAB39 induces L-OHP chemoresistance in CRC cells

**Fig. 3:**
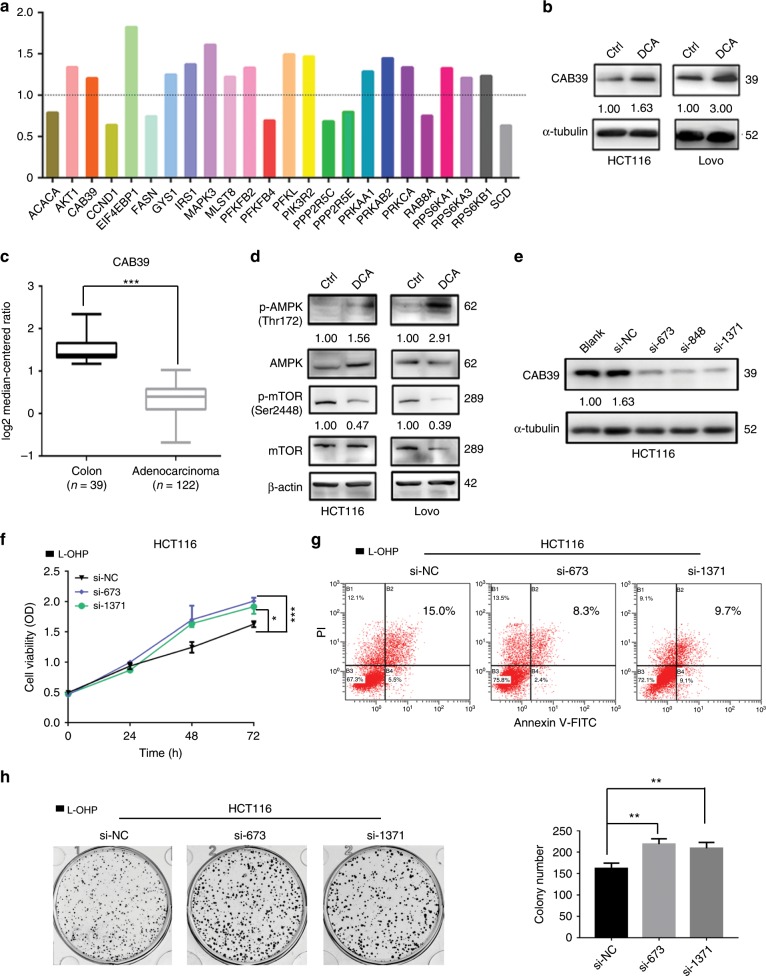
CAB39 downregulation promotes resistance of L-OHP. **a** DCA-responsive proteome in HCT-116 cells was assayed by using proteomic profiling technology, and altered expressions of proteins involved in AMPK signalling pathway were listed. **b** Expression of CAB39 in colon normal tissues and adenocarcinoma (Oncomine database). **c**, **d** The protein levels of CAB39, p-AMPK, AMPK, p-mTOR and mTOR were detected following 20 mM DCA treatment in HCT-116 and LoVo cells. **e** Expression of CAB39 protein in HCT-116 cells transfected with siCAB39 after 48 h. **f–h** HCT-116 cells were transiently transfected with siRNA, and the cells were treated with 20 μg/ml L-OHP. The cell viability was measured by CCK-8 assay. Cell apoptosis was measured by flow cytometry. Colony formation assay was determined by crystal violet staining. Experiments encompassed three replicates, *P < 0.05, **P < 0.01, ***P < 0.001.

We previously assayed the DCA-responsive proteome in HCT-116 cells and quantified 4518 proteins. Among these, 244 proteins were increased by DCA and 269 proteins were decreased by DCA.^22^ Among these apparently altered proteins, we choose those enriched in the AMPK/mTOR pathway because the AMPK/mTOR pathway is closely related to chemoresistance. The results showed that 16 proteins were upregulated, while 8 were downregulated among the proteins changed significantly after DCA treatment (Fig. 3a). Notably, CAB39, a direct upstream molecule of AMPK, was upregulated upon DCA treatment in HCT-116 and LoVo cells, which were confirmed by using Western blotting assays (Fig. 3b). Using the Oncomine database, we also found that the expression of CAB39 was significantly higher in normal epithelium tissues than in adenocarcinoma tissues (Fig. 3c). The AMPK/mTOR signal pathway was therefore next measured. We found that DCA activates the phosphorylation of AMPK and inhibits the phosphorylation of mTOR in HCT-116 and LoVo cells (Fig. 3d). Small interfering RNAs (siRNAs) were used to knockdown CAB39 expression in HCT-116 cells as shown in Fig. 3e. Using CCK-8, apoptosis and colony formation assays, we found that CAB39 knockdown increases chemoresistance of HCT-116 cells to L-OHP compared with the respective control (Fig. 3f–h) but does not affect cell proliferation and apoptosis (Supplementary Fig. 1A, 1B). In contrast, CAB39 overexpression could activate and inhibit the phosphorylation of AMPK and mTOR in HCT-116/L-OHP cells (Supplementary Fig. 1C). Moreover, we found that CAB39 overexpression decreases the survival rate, promotes apoptosis and inhibits colony formation induced by L-OHP (Supplementary Fig. 1D–1F). Finally, these data suggest that CAB39 may be involved in chemoresistance in CRC.

### DCA upregulates CAB39 through miR-107

**Fig. 4:**
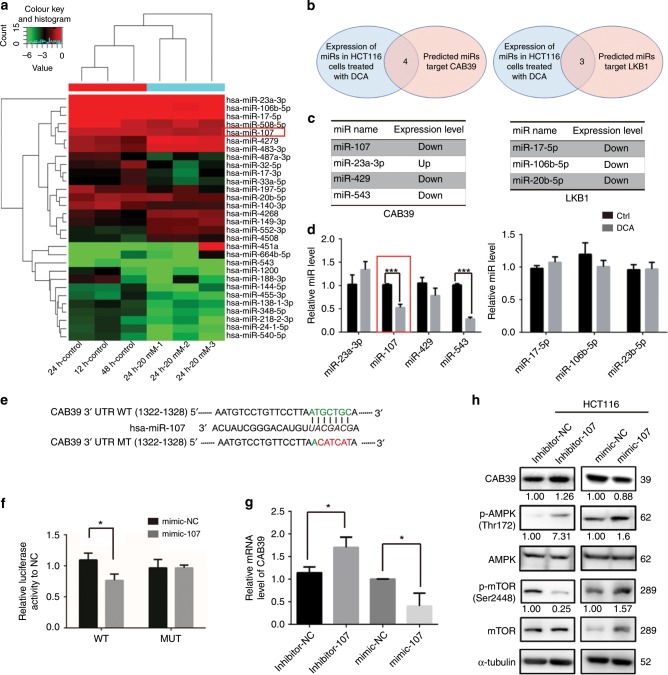
miR-107 directly targets CAB39. **a** Microarray was performed to assay miRNAs regulated by DCA (20 mM) in HCT-116 cells. **b** Schematic diagram of the protocol used to search for candidate miRNAs targeting CAB39 or LKB1 that can regulate AMPK. **c**, **d** The expressions of three and four candidate miRNAs targeting CAB39 and LKB1 in HCT-116 cells treated with or without DCA. **e** Putative binding sites of miR-107 to the 3′UTR of CAB39. The wild type and its mutant sequences are indicated in green and red, respectively. **f** HCT-116 cells were co-transfected with luciferase reporter constructs and miR-107 mimics or NC (50 nmol/L). Data were normalised to the corresponding control. **g** The mRNA expression of CAB39 after miR-107 inhibitor or mimic transfection in HCT-116 cells. **h** The protein levels of AMPK, p-AMPK, mTOR, p-mTOR and CAB39 were assayed following miR-107 mimic or inhibitor transfection in HCT-116 cells. Experiments encompassed three replicates (**d**, **f**, **g**). *P < 0.05, **P < 0.01, ***P < 0.001.

We previously determined miRNA expression profiles after DCA treatment by using a miRNA array. Candidate miRNAs that changed remarkably after DCA treatment were screened (Fig. 4a). Potential miRNA targets were predicted by TargetScan, miRWalk and miRDB. Four miRNAs were found to target the 3′UTR of CAB39 mRNA, and three could target LKB1. None of them could target STRAD after DCA treatment (Fig. 4b, c). As a result, miR-107 and miR-543 expression were decreased in DCA-treated cells (Fig. 4d). However, previous research has reported that miR-543 could target a tumour-suppressor gene, PTEN. Thus, we focused on assaying the role of miR-107 in this study. A similar result was also found in LoVo cells (Supplementary Fig. 2A).

To validate whether CAB39 is regulated directly by miR-107, wild-type (WT) or mutant-type (MT) 3′UTR of CAB39 was transfected into luciferase reporter plasmid, respectively (Fig. 4e). Reporter assays indicated that miR-107 could apparently decrease the luciferase activity of the WT CAB39 3′UTR but did not affect the activity of the MT CAB39 3′UTR. It suggested that miR-107 regulates luciferase activity through binding to the predicted site of CAB39 3′UTR (Fig. 4f). Transfection of miR-107 mimics into CRC cells (HCT-116, LoVo and HCT-8) reduced the mRNA level of CAB39, whereas miR-107 inhibitors increased the mRNA level of CAB39 (Fig. 4g and Supplementary Fig. 2B, C).

The expression levels of CAB39, AMPK, p-AMPK, mTOR and p-mTOR were also detected after transfecting miR-107 mimics or inhibitors. Protein levels of CAB39 and phosphorylated AMPK were higher, and levels of phosphorylated mTOR were lower in miR-107 inhibitor-treated cells than the control group in CRC cells (Fig. 4h and Supplementary Fig. 2D). In contrast, CAB39 and p-AMPK expression levels were decreased, and p-mTOR levels were increased in LoVo and HCT-8 cells after miR-107 mimics were transfected (Supplementary Fig. 2D). In HCT-116 cells, miR-107 mimics decreased the expression of CAB39 and activated phosphorylation of AMPK and mTOR compared with the respective controls (Fig. 4h). Collectively, the results showed that miR-107 could target the CAB39 3′UTR and suppress its expression. To confirm whether DCA regulates CAB39 expression via miR-107, a rescue assay was performed. We found that miR-107 partially abolished the upregulation effect of CAB39 after DCA treatment (Supplementary Fig. 2E). In summary, reduction of miR-107 expression induced by DCA could increase CAB39 expression.

### MiR-107 favours chemoresistance in CRC cells

**Fig. 5:**
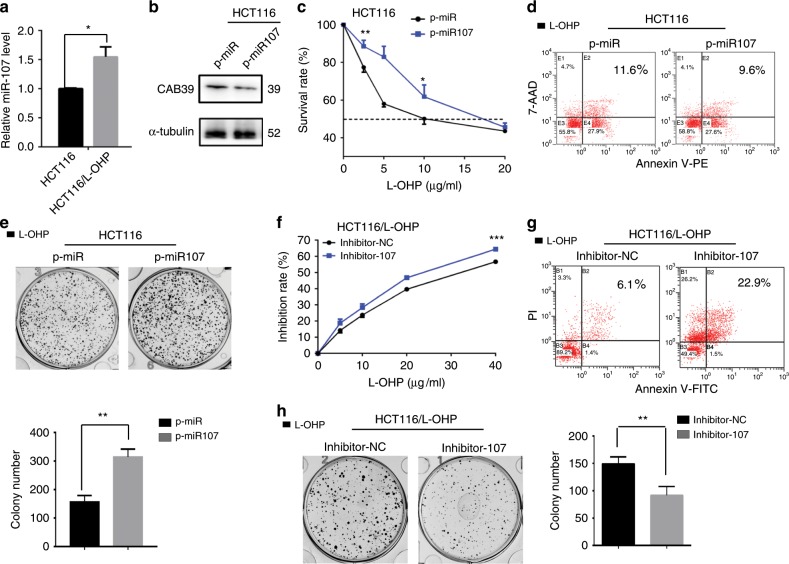
miR-107 regulates L-OHP chemosensitivity in CRC cells. **a** The expression of miR-107 in HCT-116 and HCT-116/L-OHP. **b** The expression of CAB39 protein was detected by western blot in miR-107-overexpressed HCT-116 and control cells. **c–e** Stable miR-107 overexpression and control HCT-116 cells were treated with L-OHP. The survival rate, cell apoptosis and colony formation assay were measured by CCK-8, flow cytometry and crystal violet staining. **f–h** Stable miR-107 knockdown and control HCT-116/L-OHP cells were treated with L-OHP. The survival rate, cell apoptosis and colony formation assay were measured by CCK-8, flow cytometry and crystal violet staining. The results of three independent experiments are shown. *P < 0.05, **P < 0.01, ***P < 0.001.

We analysed the expression of miR-107 in HCT-116 and HCT-116/L-OHP cells. The L-OHP-resistant CRC cells showed higher miR-107 expression than the parental CRC cells (Fig. 5a). Stable miR-107 overexpression in HCT-116 cells was established, and CAB39 expression was reduced (Fig. 5b). Using CCK-8, apoptosis and colony formation assays, we noted that overexpression of miR-107 significantly decreased L-OHP chemosensitivity in HCT-116 cells compared with the control (Fig. 5c–e). In contrast, reduction of miR-107 expression increased the inhibition rate, promoted apoptosis and inhibited colony formation induced by L-OHP (Fig. 5f–h).

In addition, we found that CAB39 overexpression decreased the survival rate, and promoted apoptosis induced by L-OHP after miR-107 mimic transfection (Supplementary Fig. 3A, B).

### MiR-107 increases L-OHP resistance in vivo

**Fig. 6:**
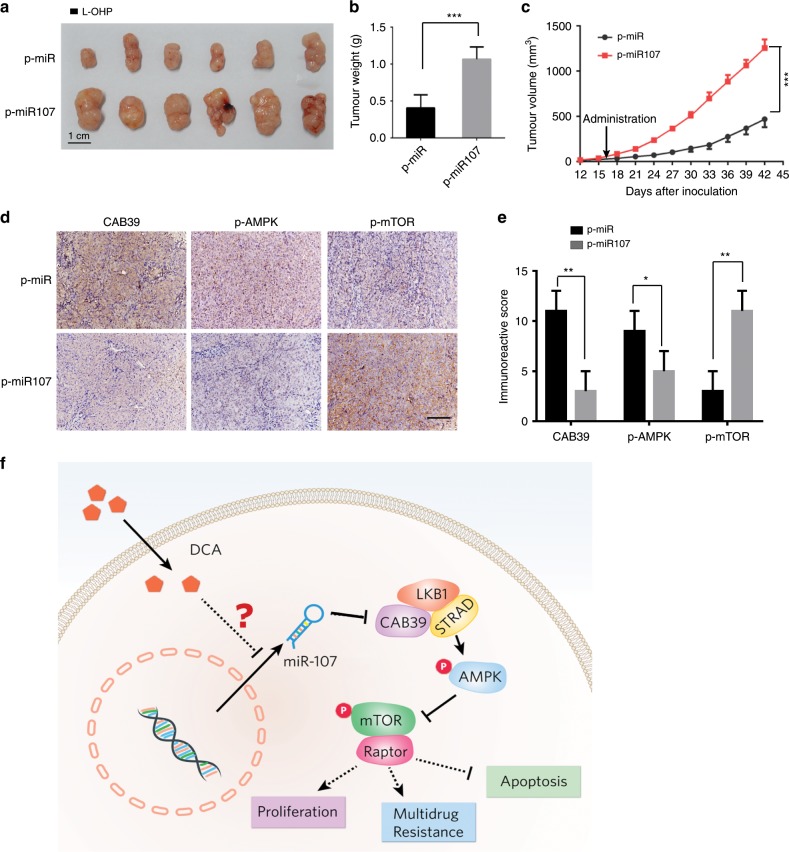
miR-107 promotes L-OHP resistance in vivo. HCT-116/miR-107-overexpressing cells and HCT-116/vector cells were injected subcutaneously. Both groups received intraperitoneal injections of L-OHP. **a** Representative photograph of tumours (scale bar: 1 cm). **b**, **c** Tumour weight and volume were measured. **d**, **e** Immunohistochemistry (IHC) detection of CAB39, p-AMPK and p-mTOR in tumour tissues. Scale bar: 100 µm. *n* = 6, *P < 0.05, **P < 0.01, ***P < 0.001. **f** A schematic diagram for illustrating the miR-107/CAB39/AMPK/mTOR pathway partially involved in the effect of DCA on increasing chemosensitivity.

To further analyse the effect of miR-107 in vivo, HCT-116/miR-107-overexpressing cells and HCT-116/vector cells were injected subcutaneously and the tumour size was measured. Both groups received intraperitoneal injections of L-OHP. We found that the tumour volumes and weights were significantly greater in the HCT-116/miR-107-overexpressing group (miR-107-OE) than those in the HCT-116/vector group (Fig. 6a–c). The expression levels of CAB39 and p-AMPK were decreased, while that of p-mTOR was increased in the miR-107-OE group (Fig. 6d). The results were confirmed by quantification analyses (Fig. 6e).

The expression of CAB39 and miR-107 was investigated in a cohort of 24 matched colorectal/normal samples. We found that CAB39 was significantly downregulated in colorectal cancer tissues compared with that in normal tissues (Supplementary Fig. 4A), which was consistent with our previous conclusion (Fig. 2b). However, no marked differences were observed between the expression of miR-107 in tumour and normal tissues (Supplementary Fig. 4B, C). Larger cohorts are required to validate the correlation between miR-107 and CAB39 expression.

## Discussion

In the clinic, chemotherapy is still one of the main treatment methods beyond surgery, radiotherapy and biological therapy. However, the development of chemoresistance constitutes the main cause of chemotherapy failure.^23^ Thus, exploring strategies for overcoming chemoresistance in CRC cells is becoming an urgent need. In this study, we found that a novel miR-107/CAB39/AMPK/mTOR signalling axis regulates L-OHP resistance in CRC cells, and that DCA can effectively increase L-OHP sensitivity partly through this signalling pathway, which may aid in the design of optimal chemotherapy strategies to increase the treatment efficacy for CRC (Fig. 6f).

The AMPK/mTOR pathway is closely related to cellular energy metabolism and is a key regulator of cell cycle arrest, apoptosis, protein and cholesterol synthesis and other important biochemical processes. Studies have shown that AMPK is a negative regulator of tumour cell proliferation;^24,25^ for example, AMPK pathway activation can inhibit the growth of tumour cells and promote their sensitivity to chemotherapy drugs.^7,26^ Moreover, mTOR pathway activation has been reported to promote tumour cell capacitation through anaerobic glycolysis, and mTOR pathway inhibitors have been demonstrated to inhibit tumour cell growth when combined with existing chemotherapeutic agents.^27^ LKB1 forms a heterotrimeric complex with ste20-related adaptor and CAB39.^10^ LKB1 tumour-suppressor activity is caused partly by AMPK-mediated inhibition of inappropriate mTOR activation.^28^ We found that DCA could upregulate CAB39, activate the AMPK pathway and inhibit the mTOR pathway. Similar to our findings, CAB39 has been shown to play key roles in inhibiting proliferation and migration.^29–31^ However, the high expression of CAB39 in hepatocellular cancer was found to be related to growth and metastasis by activating the extracellular signal-regulated kinase pathway.^32^ The reason for different CAB39 behaviours in different cancer types might be attributed to its type of activation. Our study showed that CAB39 was upregulated after DCA treatment, and CAB39 inhibition was associated with tumour cell resistance to L-OHP, suggesting that CAB39 plays an important role in regulating the chemoresistance of CRC. Moreover, we found that CAB39 expression was lower in tumour tissues than in adjacent tissues, indicating that CAB39 acts as a tumour suppressor in CRC.

miRNAs are known to play diverse roles in human tumours, as well as in the development of chemoresistance, depending on their context in different tissues. miR-107 directly targeted CAB39 and subsequently affected the AMPK/mTOR pathway, eventually leading to chemoresistance, as summarised in the schematic diagram in Fig. 6. Although miR-107 has been reported to have different effects in several types of cancers,^20,21,33^ our research indicated that miR-107 accelerates proliferation of CRC cells treated with L-OHP in vitro and in vivo, suggesting that miR-107 promotes chemoresistance in CRC.

DCA is a traditional small-molecule agent used to treat lactic acidosis in the clinic and has proven to be safe. In recent years, DCA was found to inhibit the growth of multiple malignancies such as human breast cancer, non-small-cell adenocarcinoma and glioblastoma cell lines; DCA also inhibits endometrial cancer, neuroblastoma and other malignant tumours.^34–36^ When combined with etoposide and cisplatin, carboplatin, platinum and its metabolite JM118, DCA was found to have significantly increased the antitumour effects.^37–39^ There are two main mechanisms accounting for DCA’s anticancer effect: 1) reversal of the Warburg effect to inhibit cancer cell proliferation and activate caspase-mediated apoptosis and 2) the oxidative elimination of lactic acid by the co-buffer action of pyruvate and hydrogen ions in the mitochondrial matrix.^40^

Taken together, we disclose that miR-107/CAB39/AMPK/mTOR signal pathway is partially involved in the effect of DCA on increasing chemosensitivity, thus providing a promising target for CRC treatment.

## Supplementary information

supplementary figure

## Data Availability

miRNA microarray data were submitted to GEO database (GSE125309). The authors declare that the data supporting the findings of this study are available within the paper.
