# Supplementary material for: MiR-107 confers chemoresistance to colorectal cancer by targeting calcium-binding protein 39
Source: Br J Cancer. 2020 Jan 10;122(5):705–14. doi: 10.1038/s41416-019-0703-3 (PMC7054533; doi:10.1038/s41416-019-0703-3)
Supplement: Supplementary file 1 — supplementary figure [file 41416_2019_703_MOESM1_ESM.pdf]

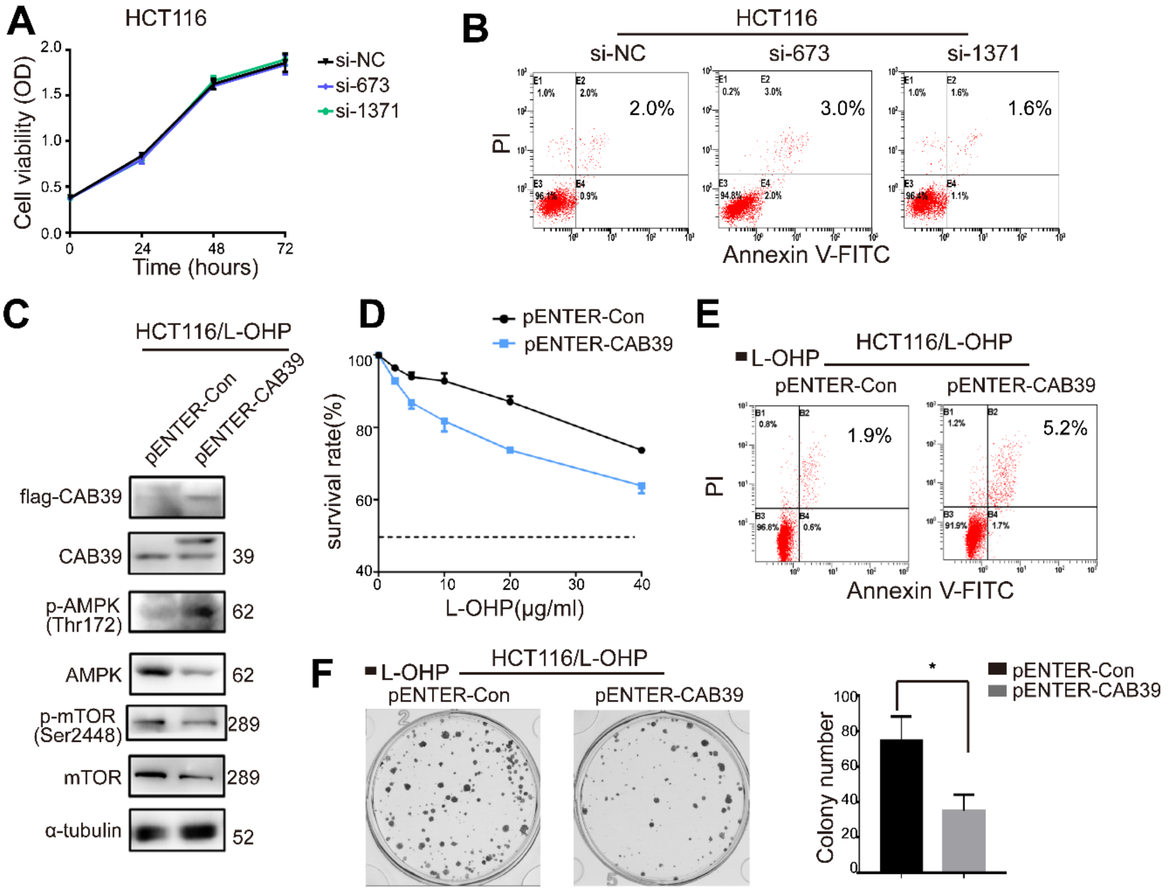

Supplementary Figure 1. CAB39 overexpression increases L-OHP chemosensitivity

(A-B) HCT116 cells were transiently transfected with siCAB39. The cell viability was measured by CCK8 assay. Cell apoptosis was measured by flowcytometry. (C) The expressions of AMPK, p-AMPK, mTOR, p-mTOR, CAB39 were detected following CAB39 plasmid transfection. (D) HCT116/L-OHP cells were transfected with CAB39 overexpression plasmid, and then treated with different concentrations of L-OHP. The survival rate was measured by CCK8 assay, (E-F) HCT116/L-OHP cells were transfected with CAB39 overexpression plasmid, and then treated with L-OHP. Cell apoptosis was measured by flowcytometry. Colony formation assay was determined by crystal violet staining. Experiments encompassed 3 replicates, *P<0.05, **P<0.01, ***P<0.001.

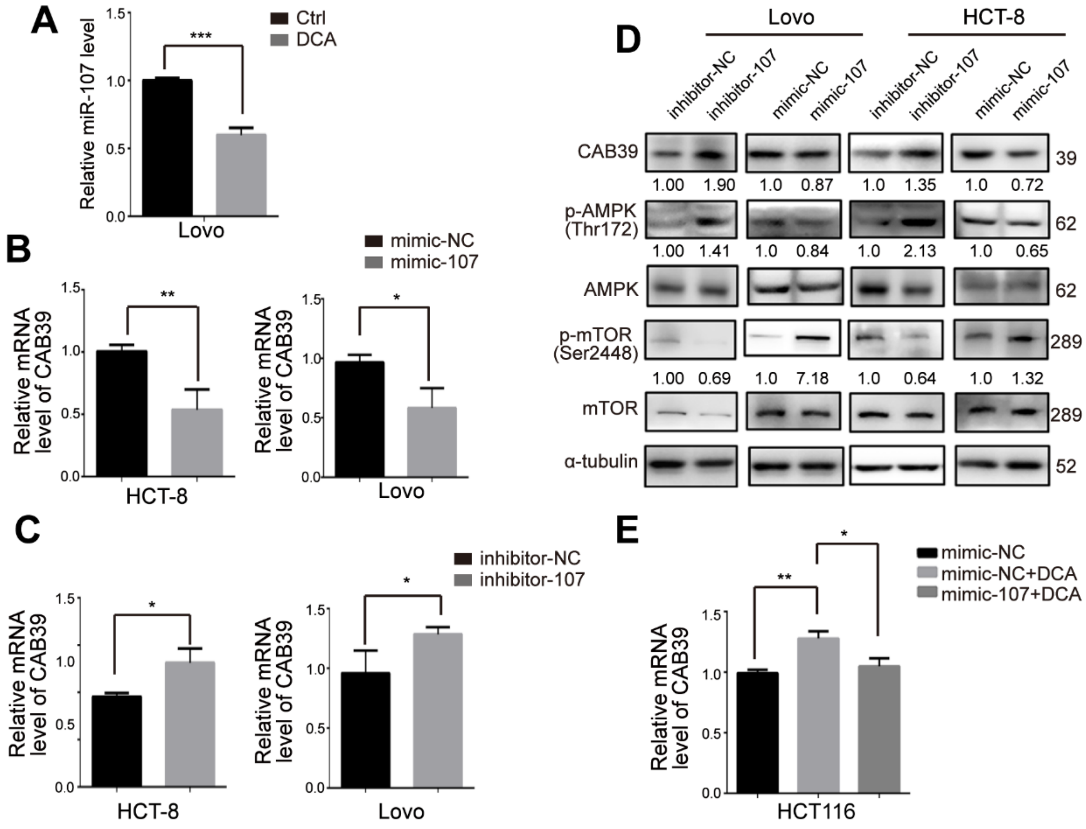

Supplementary Figure 2. miR-107 regulates AMPK/mTOR signaling pathway through CAB39

(A)The expression of miR-107 after DCA treatment in LOVO cells was detected by quantative real-time PCR. (B) The mRNA level of CAB39 after miR-107 mimic transfection in HCT-8 and Lovo cells. (C) The mRNA level of CAB39 after miR-107 inhibitor transfection in HCT-8 and Lovo cells. (D) The protein levels of AMPK, p-AMPK, mTOR, p-mTOR, CAB39 were assayed following miR-107 mimic or inhibitor transfection in HCT-8 and Lovo cells. (E) HCT116 cells were transfected with miR-107 mimics or corresponding control (mimic-NC), and then treated with DCA for 24 h before RNA extraction. The mRNA level of CAB39 was detected by quantative real-time PCR. Experiments encompassed 3 replicates. *P<0.05, **P<0.01, ***P<0.001.

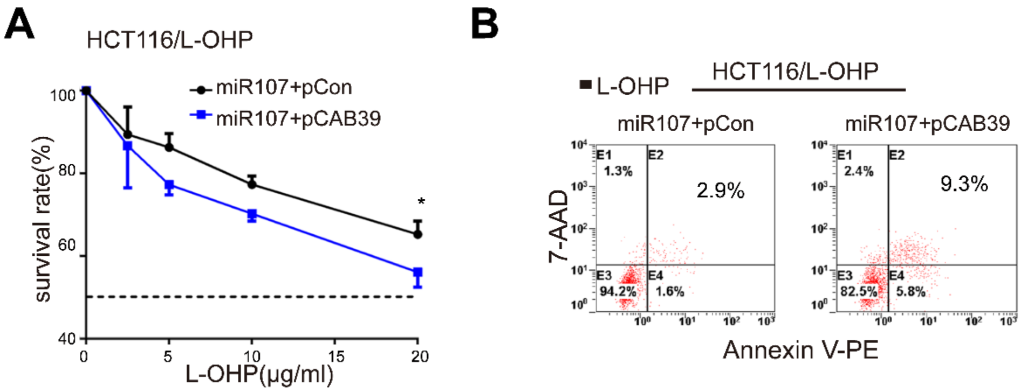

Supplementary Figure 3. CAB39 overexpression partially antagonizes the effect of miR-107 enhancing chemoresistance in HCT116/L-OHP cells

(A) HCT116/L-OHP cells were transfected with CAB39/Con plasmid and miR-107 mimics, and then treated with different concentrations of L-OHP. The survival rate was measured by CCK8 assay. (B) HCT116/L-OHP cells were transfected with CAB39/Con plasmid and miR-107 mimics, and then treated with L-OHP. Cell apoptosis was measured by flowcytometry. Experiments encompassed 3 replicates, *P<0.05, **P<0.01, ***P<0.001.

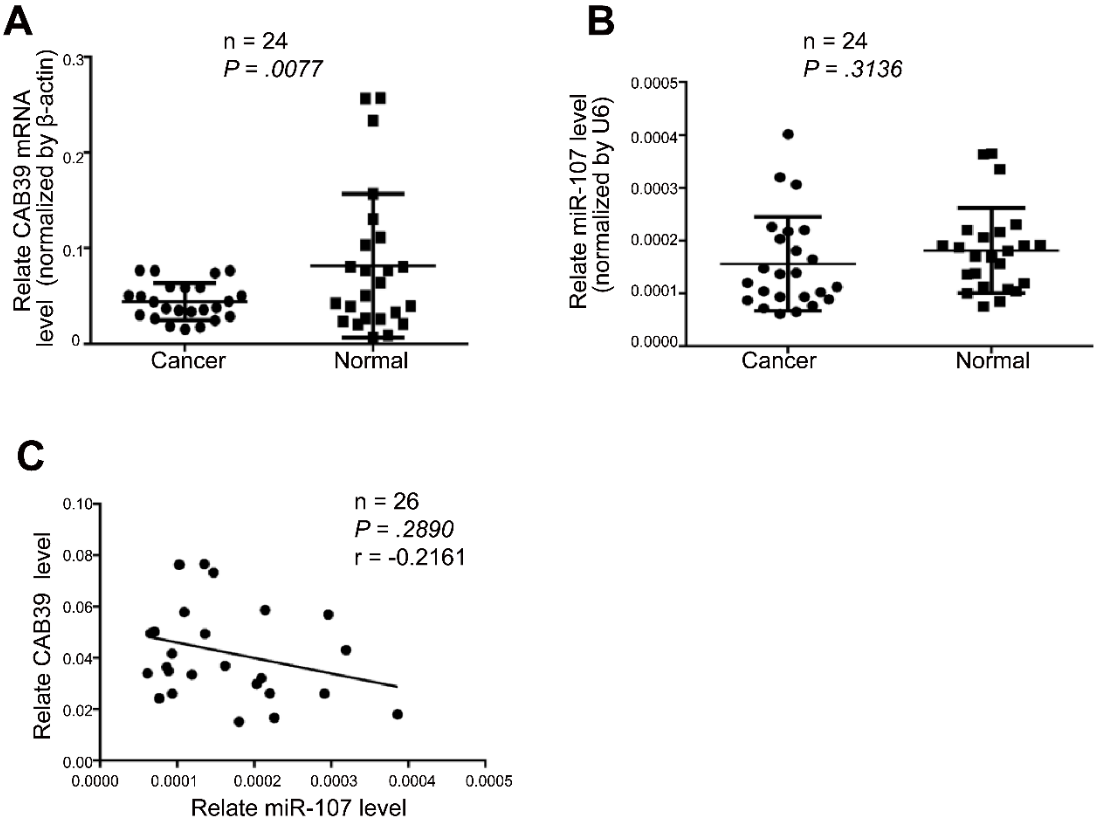

Supplementary Figure 4. CAB39 level is inversely correlated with miR-107 level

(A-B) Relative levels of miR-107 and CAB39 in matched human colon cancer/normal tissues.

(C) The correlation between the expression of miR-107 and CAB39 was determined using linear regression analysis (n = 26, r = -0.2161, *P*=0.2890)
